# Bioactive compounds and exercise in aging and neurodegeneration: mechanistic insights from the gut–brain–metabolic axis

**DOI:** 10.3389/fnut.2026.1781176

**Published:** 2026-04-08

**Authors:** Zhan-Tao Feng, Yue-Yan Zhang, Chun-Xiang Xue

**Affiliations:** 1 College of Sports and Health, Shandong Sport University, Jinan, Shandong, China; 2 Qufu Normal University Affiliated Experimental School, Rizhao, Shandong, China; 3 Department of Sports and Leisure, Dongshin University, Naju, Republic of Korea

**Keywords:** aging, bioactive compounds, exercise, memory, neurodegenerative diseases

## Abstract

Aging and neurodegenerative disorders are associated with impaired hippocampal plasticity, yet existing literature largely examines exercise, nutrition, or metabolic regulation in isolation. This review synthesizes emerging evidence supporting an integrative neuro-nutritional-metabolic framework in which bioactive compounds and physical exercise converge to modulate hippocampal neurogenesis, synaptic plasticity, and cognitive resilience. Recent investigative efforts elucidate the neuro-nutritional-metabolic axis as a pivotal interface that integrates bioactive compounds derived from diet, systemic metabolic processes, and neuronal functionality. In this review, the term ‘neuro-nutritional-metabolic axis’ refers to an integrative framework describing the bidirectional interactions among dietary bioactive compounds, systemic metabolic regulation, and central nervous system plasticity. This concept extends established models such as the microbiota–gut–brain axis and muscle–brain communication by emphasizing their convergence on metabolic and neurotrophic signaling pathways relevant to hippocampal function. Simultaneously, physical exercise is acknowledged as a significant modulator of neurotrophic signaling pathways, mitochondrial performance, and neuroinflammatory responses. This review synthesizes mechanistic evidence derived predominantly from preclinical studies alongside emerging but comparatively limited clinical findings to evaluate how bioactive compounds and physical exercise interact to influence hippocampal plasticity and cognitive function. We examine the convergence of these interventions on essential molecular pathways, as well as antioxidant and anti-inflammatory cascades, to facilitate neuronal survival, synaptic reorganization, and cognitive resilience. Moreover, we investigate their potential to mitigate metabolic dysfunction, oxidative stress, and chronic inflammation, which are pivotal factors contributing to cognitive deterioration in the context of aging and neurodegenerative conditions such as Alzheimer’s and Parkinson’s disease. Comprehending these synergistic interactions lays the groundwork for formulating tailored, multimodal interventions that specifically address the neuro-nutritional-metabolic axis to enhance memory retention, optimize learning processes, and support cognitive resilience and may contribute to the modulation of risk factors associated with neurodegenerative conditions.

## 1 Introduction

Age-associated cognitive decline and neurodegenerative disorders (*NDs*) such as Alzheimer’s disease (AD) and Parkinson’s disease (PD) are delineated by progressive perturbations in hippocampal circuitry, which constitutes the neural substrate for memory and learning processes. Contrary to longstanding beliefs suggesting that the aging brain possesses a constrained capacity for structural reorganization, an increasing body of evidence indicates that adult hippocampal neurogenesis and synaptic plasticity remain amenable to modification throughout the lifespan and can be profoundly influenced by lifestyle determinants, particularly bioactive compounds derived from dietary sources and physical exercise (1, 2). This rapidly burgeoning corpus of research underscores the presence of a neuro-nutritional-metabolic axis through which nutrients, systemic metabolic processes, immune modulators, and exercise-induced factors interact to regulate neuronal stem cell dynamics, neurotrophic signaling pathways, mitochondrial integrity, and synaptic reconfiguration. In this review, the term ‘gut–brain–metabolic axis’ is used as an integrative conceptual framework that extends the established gut–brain axis to include systemic metabolic regulation and exercise-induced peripheral signaling (e.g., myokines and metabolic hormones). This framework reflects the bidirectional interactions among gut microbiota, peripheral metabolism, and central nervous system plasticity that collectively influence cognitive function in aging and neurodegenerative conditions.

Nonetheless, the extant literature continues to exhibit a significant degree of fragmentation: the majority of scholarly articles predominantly concentrate on either physical activity as an independent intervention or on discrete categories of bioactive molecules, including polyphenols, omega-3 fatty acids, vitamins, or carotenoids (3, 4). Although these studies furnish critical mechanistic insights, none provide a comprehensive and systematic examination of how these elements coalesce, interact, or collaboratively modulate hippocampal neurogenesis within the frameworks of aging or *NDs*. Current reviews either explore the implications of exercise on neuroplasticity, the neuroprotective efficacy of particular nutrient categories, or general lifestyle interventions pertaining to cognitive aging; however, they fall short of delivering an in-depth, mechanism-focused synthesis of how physical activity and nutritional bioactives collectively influence metabolic, immune, endocrine, and epigenetic pathways that affect cerebral function (5, 6).

Moreover, recent advancements, including the characterization of irisin as a crucial mediator that connects skeletal muscle activity to hippocampal brain-derived neurotrophic factor (BDNF) signaling (7), as well as the elucidation of metabolite-mediated gut–brain interactions influenced by polyphenols and physical exercise, have yet to be integrated into a cohesive conceptual framework. These developments have transpired predominantly within the past five years, rendering a contemporary synthesis particularly relevant. No existing review offers a thorough, mechanistic, and clinically pertinent amalgamation of the neuro-nutritional-metabolic axis, its molecular mediators, and their prospective role in augmenting hippocampal neurogenesis and cognitive learning in the context of aging or *NDs*. The present study endeavors to address this specific deficiency. A meticulous appraisal of the extant literature reveals both notable strengths and inherent limitations. Preclinical models consistently elucidate that physical exercise might augment the levels of BDNF within the hippocampus (see Section 4), enhance mitochondrial functionality, mitigates neuroinflammation, and promotes neurogenesis; concurrently, various bioactive compounds such as resveratrol, curcumin, EGCG, DHA, and astaxanthin exhibit analogous effects via antioxidant, anti-inflammatory, mitochondrial, and epigenetic mechanisms (8, 9). Importantly, a number of these compounds have been shown to enhance neurotrophic signaling or heighten neuronal responsiveness to BDNF, thereby implying a molecular basis for potential synergistic interactions. Nonetheless, in spite of the compelling mechanistic parallels, the existing literature reveals a paucity of studies that have explored combined interventions; furthermore, those that have been conducted exhibit considerable variability in terms of exercise modalities, bioactive compound dosages and formulations, treatment durations, and outcome measures, thus complicating comparative analyses and constraining translational applicability.

Human research, while encouraging, continues to be limited in scope. Preliminary clinical investigations indicate that sustained aerobic exercise contributes to enhancements in hippocampal volume and cognitive performance; concurrently, dietary patterns abundant in polyphenols or omega-3 fatty acids are associated with more favorable cognitive aging trajectories. Nonetheless, intervention studies that integrate multiple approaches are infrequent, typically characterized by brief durations, and seldom incorporate mechanistic biomarkers such as neurotrophic factors, exosomal microRNA, or hippocampal imaging indicators sensitive to structural plasticity (10, 11). Notable tensions and unresolved inquiries arise from the existing literature. For instance, certain antioxidants may inhibit exercise-induced hormetic responses when administered in elevated doses, suggesting that synergistic effects are not inherently assured, but rather contingent upon timing and dosage. Numerous promising bioactive compounds experience challenges related to low bioavailability, prompting inquiries about the most effective formulations when utilized in conjunction with exercise. While the mechanistic convergence of exercise and bioactive compounds is strongly supported by preclinical models, clinical evidence directly examining combined interventions in aging and neurodegenerative populations remains comparatively limited and heterogeneous.

Moreover, the gut microbiome demonstrates a notable sensitivity to both physical exercise and polyphenolic compounds; however, existing research seldom investigates the metabolites derived from the microbiome in relation to outcomes within the hippocampus. These deficiencies highlight the necessity for comprehensive mechanistic frameworks and forthcoming trials employing multimodal biomarker methodologies. Taken together, the extant evidence compellingly indicates that modulating the neuro-nutritional-metabolic axis through the synergistic use of bioactive compounds and regimented physical exercise may present a formidable approach to augmenting hippocampal neurogenesis, synaptic plasticity, memory retention, and learning capabilities in aging populations and those afflicted by neurodegenerative conditions. The principal insights derived from contemporary research and practical applications can be summarized as follows: (i) the mechanistic convergence between factors induced by exercise and signals derived from nutrients establishes a robust biological justification for their synergy; (ii) although the evidence from animal studies is substantial, human data necessitate larger-scale, longitudinal, and biomarker-integrated trials to ascertain clinical effectiveness; (iii) personalized approaches are essential, as responses vary with genotype, metabolic state, microbiome composition, and comorbidities; and (iv) until more definitive data emerge, a practical recommendation is to combine sustained moderate-to-vigorous exercise with diets rich in polyphenol-containing fruits, vegetables, teas, omega-3 sources, and anti-inflammatory nutrients, while avoiding excessive antioxidant supplementation that might disrupt adaptive responses.

To enhance conceptual clarity, the mechanisms discussed in this review are organized into three hierarchical domains. Primary mechanisms include neurotrophic signaling and mitochondrial-metabolic regulation, which directly govern neuronal survival, synaptic plasticity, and adult hippocampal neurogenesis. Secondary mechanisms encompass modulation of oxidative balance and neuroinflammatory tone, which shape the cellular environment that permits or constrains plasticity-related processes. Contextual modulators include gut microbiota-derived metabolites, endocrine signaling, and exercise-induced peripheral factors that influence the magnitude and persistence of these effects. This framework is used throughout the manuscript to distinguish central drivers of hippocampal plasticity from supportive physiological processes.

## 2 The neuro-gut-metabolic axis

Bioactive compounds serve as pivotal stimulators of the gut–brain–metabolic axis by influencing the composition of gut microbiota, the generation of microbial-derived metabolites, and the signaling pathways related to host metabolism. Through their interactions with intestinal epithelial cells, immune response pathways, and neuroendocrine signaling mechanisms, these compounds establish a bidirectional communicative link between the gastrointestinal tract and the central nervous system. The activation of this axis results in enhancements in energy metabolism, a decrease in systemic and neuroinflammation, and an augmentation of mitochondrial functionality. In conjunction with physical exercise, bioactive compounds facilitate the processes of hippocampal neurogenesis and synaptic plasticity, thus leading to improvements in cognitive functions such as memory and learning. This modulation of the gut–brain–metabolic axis, driven by activation, holds particular significance in the context of aging and neurodegenerative disorders, wherein metabolic dysregulation and compromised neuroplasticity play a role in the deterioration of cognitive abilities. This axis encompasses a variety of interdependent elements, including nutrient consumption and assimilation, metabolites derived from the gut microbiome, peripheral metabolic organs such as skeletal muscle and liver, endocrine hormones, immune mediators, and central neural networks integral to cognitive processes such as learning and memory (12, 13). Bioactive nutritional compounds exert influence over oxidative equilibrium, mitochondrial efficiency, inflammatory status, lipid composition, and epigenetic modifications. These biochemical and physiological alterations culminate in adaptations of brain plasticity by impacting neurotrophic signaling pathways, mitochondrial biogenesis, neuronal viability, and neurogenesis within the adult hippocampus. Engaging in physical exercise acts as a complementary modulator within this axis by influencing energy metabolism, myokine signaling, insulin sensitivity, and metabolic adaptability (14, 15).

**Figure 1:**
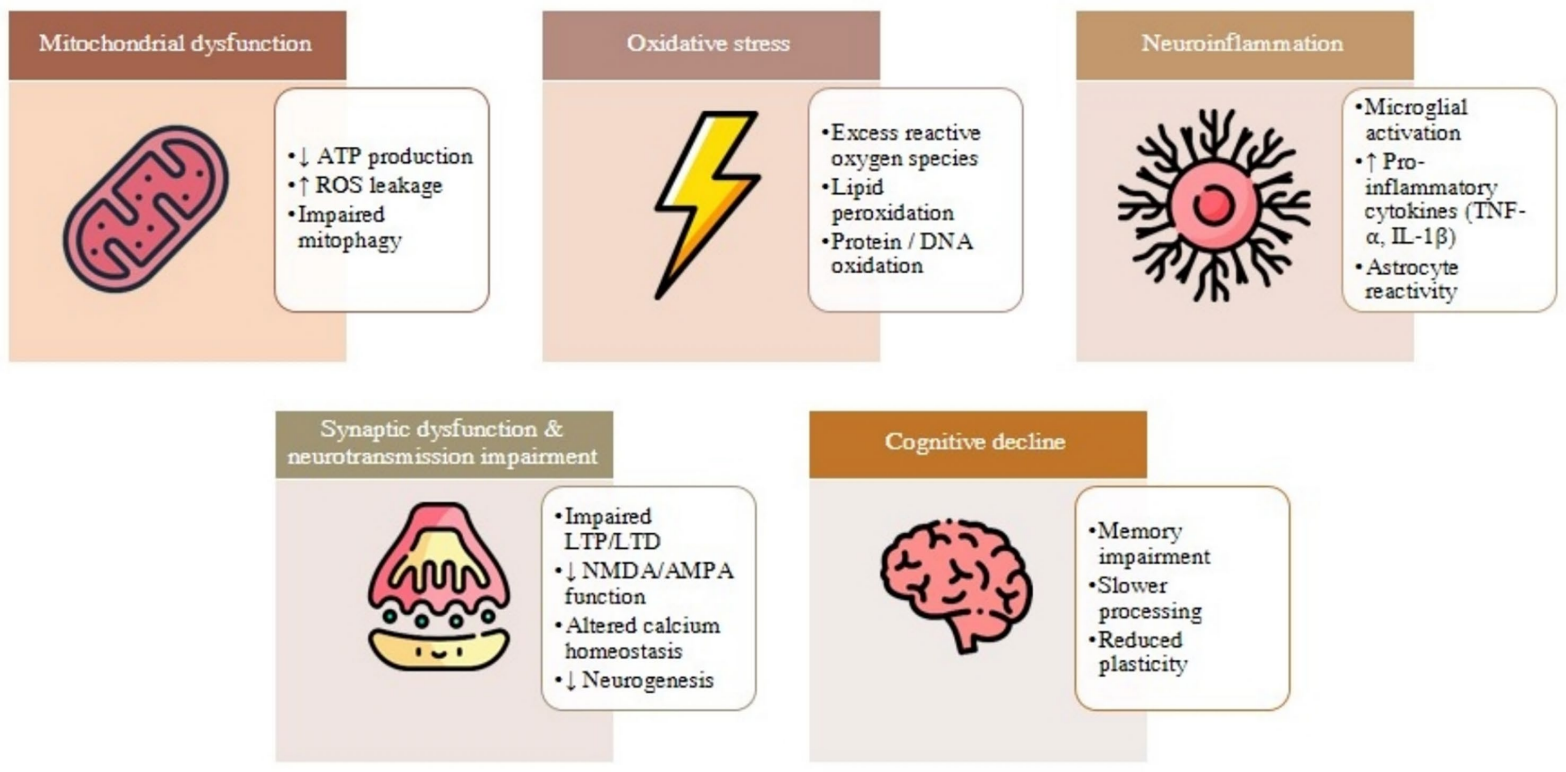
The neuro-nutritional-metabolic axis supporting hippocampal function. This diagram illustrates how nutritional bioactive compounds and systemic metabolic factors independently feed into the brain to support hippocampal neurogenesis, synaptic plasticity, and cognitive function. Key pathways include enhanced mitochondrial activity, improved insulin sensitivity, reduced oxidative stress and inflammation, and increased BDNF/CREB signaling. Together, these inputs converge to promote a resilient and metabolically supported hippocampus.

Metabolism and nutrition exert profound and extensive effects on cognitive functioning through various biochemical pathways. The brain relies on a continuous influx of glucose, ketone bodies, lipids, amino acids, and micronutrients to facilitate neurotransmission, synaptic plasticity, and mitochondrial adenosine triphosphate synthesis. Nutritional status significantly influences the operational dynamics of critical regulatory systems that govern cellular resilience, synaptic efficacy, neurogenesis, and neuronal adaptability (16, 17). Numerous bioactive compounds traverse the blood–brain barrier or exert their effects indirectly via peripheral metabolic and immune mechanisms, as well as through metabolites derived from gut microbiota, such as short-chain fatty acids. Physical exercise augments these biological processes by enhancing cerebral blood circulation, stimulating mitochondrial biogenesis, increasing the expression of neurotrophic factors, and modulating the oxidative-reductive environment. Collectively, these interactions underscore the reciprocal relationship inherent in the neuro-nutritional-metabolic axis, wherein peripheral metabolic conditions continuously modulate central nervous system plasticity, while neural activity reciprocally influences metabolic requirements and nutrient utilization. Aging and *NDs* unravel this intricate axis through a myriad of mechanistic pathways. The metabolic deterioration associated with aging diminishes the dialog between external nutrient signals and the brain’s adaptability (18). The aging hippocampus grows increasingly unresponsive to neurotrophic cues, while the phenomenon of inflammaging stifles neurogenesis and the restoration of synapses. Neurodegenerative ailments like Alzheimer’s further jeopardize this axis by hindering mitochondrial oxidative phosphorylation, heightening oxidative stress, skewing glucose and lipid metabolism, and fostering gut dysbiosis that disrupts the creation of neuroprotective microbial metabolites (19, 20). Diminished physical activity, often seen in the aging process, worsens these irregularities by curtailing myokine production, weakening antioxidant defenses, disrupting glucose balance, and intensifying metabolic inflexibility. Consequently, the dynamic interrelation between nutrition, metabolism, and brain adaptability becomes disjointed, hastening cognitive decline and diminishing resistance to neurodegenerative processes. Understanding how to restore or optimize this axis through targeted bioactive compounds and structured exercise represents a promising strategy for mitigating age-related cognitive impairment and slowing the progression of neurodegenerative diseases (Figure 1).

## 3 Role of bioactive compounds in hippocampal function

Bioactive compounds have emerged as formidable influencers of hippocampal neurogenesis, synaptic adaptability, and cognitive fortitude. Among these, polyphenols, omega-3 fatty acids, carotenoids, vitamins, and an array of other functional nutrients wield multifaceted effects that converge on essential molecular pathways vital for hippocampal vitality. Their influence becomes particularly significant in the realms of aging and *NDs*, where oxidative stress, neuroinflammation, mitochondrial dysfunction, and disrupted neurotrophic signaling render the hippocampus susceptible to damage. Importantly, the beneficial effects of dietary bioactives described here should be interpreted within a hormetic framework, as excessively high-dose antioxidant supplementation may attenuate adaptive responses to exercise.

Polyphenols showcase remarkable neuroprotective qualities. These compounds amplify memory and learning by elevating BDNF levels and fine-tuning CREB-dependent transcription, thus fostering long-term potentiation (LTP) (21, 22). Flavonoids also possess the ability to rejuvenate mitochondrial performance and mitigate neuroinflammation via the Nrf2–ARE antioxidant pathway and the suppression of NF-κB signaling (23). Resveratrol, a stilbene polyphenol, offers additional advantages by engaging SIRT1 and AMP-activated Protein Kinase (AMPK) pathways, optimizing neuronal energy metabolism, enhancing adult hippocampal neurogenesis, and dampening microglial activation (24, 25). Significantly, these mechanisms engage in synergistic interactions with physical exercise, which concurrently targets intersecting biological pathways such as BDNF and AMPK. Omega-3 polyunsaturated fatty acids serve as essential structural constituents of neuronal membranes and act as modulators of synaptic signaling processes. Docosahexaenoic acid (DHA) is associated with hippocampal functionality by improving membrane fluidity, facilitating the formation of dendritic spines, and promoting the synthesis of neurotrophins (26). Moreover, omega-3 fatty acids mitigate neuroinflammatory responses through resolvin-mediated pathways and diminish oxidative stress by enhancing mitochondrial respiration. In the context of aging and *NDs* such as AD, DHA supplementation has been demonstrated to reduce amyloidogenic processes, regulate glucose utilization within the hippocampus, and sustain cognitive performance (27).

Carotenoids and vitamins, including vitamins C, D, and E, exert significant effects on the integrity of the hippocampus through various complex biochemical mechanisms. Lutein and zeaxanthin preferentially accumulate in neural tissues and demonstrate a strong correlation with increased hippocampal volume and cognitive performance in the elderly population (28). Astaxanthin possesses remarkable antioxidant and anti-inflammatory properties, which alleviate oxidative stress in the hippocampus and modulate the PI3K/Akt and Nrf2 signaling pathways (29). Vitamins E and C play a crucial role in safeguarding neuronal membranes against lipid peroxidation (30), whereas vitamin D is instrumental in regulating calcium homeostasis and diminishing microglial activation, thereby enhancing neurogenesis and synaptic plasticity (31). Other functional nutrients, encompassing amino acids, minerals, and gut-derived metabolites such as short-chain fatty acids, exert influence on hippocampal functionality via neurochemical and metabolic pathways. Zinc assumes a pivotal role in facilitating synaptic transmission and plasticity within the mossy fiber pathway, while magnesium augments NMDAR-mediated signaling, which is requisite for the processes of learning and memory (32). The availability of tryptophan modulates serotonin synthesis, a process that is indispensable for adult neurogenesis and resilience to stress. Concurrently, gut microbial metabolites have been demonstrated to regulate microglial maturation, permeability of the blood–brain barrier, and neurogenesis within the hippocampus, thereby establishing a connection between nutrition and the homeostasis of the central nervous system.

In summary, these bioactive constituents collectively exert a synergistic effect on the health of the hippocampus by addressing oxidative stress, inflammation, mitochondrial dynamics, synaptic communication, neurotrophic pathways, and epigenetic modulation. Their ability to influence these mechanisms underscores the therapeutic promise of amalgamating neuro-nutritional interventions with physical exercise, which engages numerous overlapping molecular pathways. This intersection provides a robust justification for focusing on the neuro-nutritional-metabolic continuum to enhance cognitive capabilities and mitigate the neurobiological ramifications associated with aging and *NDs*.

## 4 Physical exercise and cognitive health

The correlation between physical exercise and cognitive health is substantial and intricate, encompassing exercise type and intensity, impacts on hippocampal neurogenesis and synaptic plasticity, as well as well-defined molecular pathways, all of which enhance memory and learning capabilities (15). Various forms of exercise provoke overlapping yet distinct systemic and central responses. Aerobic exercise consistently augments cerebral blood circulation, fosters angiogenesis, and over the course of weeks to months is correlated with increases in hippocampal volume and enhancements in episodic memory among older adults (15, 33). Resistance training is related to executive functioning and processing velocity, and is particularly effective in maintaining frontal-hippocampal networks during aging, with sessions of moderate intensity conducted biweekly demonstrating positive outcomes in multiple studies (34). High-Intensity Interval Training (HIIT) elicits significant acute metabolic and neurotrophic responses; furthermore, when implemented over an extended duration, it has been empirically demonstrated to enhance working memory and cognitive flexibility, likely attributable to substantial elevations in systemic exerkines and metabolic stressors that serve as signals to the brain (34, 35). Consequently, the selection of modality and intensity should be reflective of the specific cognitive domain being targeted, the baseline fitness levels and comorbid conditions of the population, as well as their overall tolerability. At the cellular and circuitous level, physical exercise facilitates adult hippocampal neurogenesis, augments dendritic complexity and spine density, and reinforces LTP, collectively contributing to improvements in pattern separation and memory encoding (36, 37). Research involving rodent models consistently indicates that voluntary wheel running or treadmill training enhances the proliferation of neural progenitor cells, fosters the survival and maturation of newly formed granule neurons, and ameliorates behaviors dependent on hippocampal function.

Human empirical evidence provides support for concordant effects: longitudinal exercise interventions have been demonstrated to augment hippocampal volume, enhance performance on memory assessments that are sensitive to hippocampal functionality, and elevate circulating levels of BDNF across numerous investigations (2, 38). Notably, the timing and duration of these exercise regimens are critical: prolonged interventions yield more enduring structural and cognitive alterations than isolated sessions or very brief programs. Mechanistically, various established molecular pathways facilitate the advantageous outcomes of exercise on hippocampal plasticity. BDNF serves as a pivotal mediator: physical activity elevates BDNF expression within both the hippocampus and peripheral systems, and BDNF, through its high-affinity receptor TrkB, initiates downstream signaling cascades that are vital for neuronal survival, synaptic potentiation, and neurogenesis (36, 39).

The interaction between BDNF and TrkB initiates three primary intracellular signaling cascades: the PI3K/Akt pathway, the Ras/ERK pathway, and the PLCγ pathway, which collectively promote LTP and structural reconfiguration (39, 40). The PI3K/Akt signaling axis also intersects with mTOR, a pivotal regulator of translational processes; mTOR-mediated protein synthesis is essential for the consolidation of synaptic modifications and the formation of dendritic spines that constitute the basis of long-term memory (40). An additional metabolic sensing pathway encompasses AMPK and its downstream coactivator PGC-1α. Physical exercise stimulates AMPK activation in both skeletal muscle and the brain in response to metabolic stress, subsequently leading to the upregulation of PGC-1α, which promotes mitochondrial biogenesis and enhances oxidative metabolism (41). PGC-1α serves as the precursor for the myokine irisin; irisin is capable of traversing the blood–brain barrier or signaling to the brain and has been associated with the upregulation of hippocampal BDNF expression, thereby establishing a connection between peripheral energy metabolism and central neurotrophic support (42, 43).

Although experimental studies support a mechanistic link between the PGC-1α/FNDC5/irisin pathway and hippocampal BDNF regulation, human findings remain inconsistent, in part due to methodological differences and ongoing concerns regarding assay specificity and reliability in the measurement of circulating irisin.

Therefore, the AMPK/PGC-1α signaling pathway not only augments neuronal energetic resilience but also establishes a mechanistic link between systemic metabolic adaptations resulting from exercise and localized trophic support within the hippocampus. The activation of these pathways induced by exercise is contingent upon contextual factors and is influenced by variables such as age, sex, baseline metabolic health, and nutritional status. For instance, diminished insulin sensitivity or mitochondrial dysfunction associated with aging may attenuate the AMPK/PGC-1α responses and impede the induction of BDNF, thereby elucidating the reduced benefits observed in certain older or metabolically compromised populations (38, 40). Furthermore, excessive antioxidant supplementation may paradoxically inhibit the exercise-induced redox signaling that is essential for the adaptive upregulation of PGC-1α and BDNF, thereby demonstrating the hormetic nature of exercise stimuli. Collectively, the existing literature substantiates a model in which appropriately calibrated exercise activates the BDNF/TrkB, PI3K/Akt/mTOR, and AMPK/PGC-1α networks, thereby facilitating enhancements in hippocampal neurogenesis, synaptic plasticity, and cognitive function (36, 40). Rather than functioning solely as direct free-radical scavengers, ROS also act as essential signaling molecules that regulate cellular adaptation, mitochondrial biogenesis, and endogenous antioxidant defenses. Consequently, excessive antioxidant supplementation may disrupt physiological redox homeostasis and attenuate beneficial hormetic responses, particularly in the context of exercise-induced stress signaling. This hormesis-based framework is important for interpreting the role of dietary bioactives discussed in later sections, as their beneficial effects are increasingly understood to arise from modulation of redox-sensitive signaling pathways (e.g., Nrf2, NF-κB, and mitochondrial stress responses) rather than nonspecific antioxidant activity. Accordingly, the efficacy of polyphenols and related compounds should be viewed as context-dependent and dose-sensitive, supporting adaptive resilience rather than simply suppressing oxidative processes. Integrating this perspective helps reconcile apparent inconsistencies in the literature and avoids internal contradiction between caution regarding excessive antioxidant supplementation and the proposed benefits of dietary bioactives. This consideration is revisited in later sections discussing dietary bioactive compounds, where distinctions are made between pharmacological-dose antioxidant supplementation and physiologically regulated exposure from whole-food sources.

## 5 Synergistic effects of bioactive compounds and exercise in aging

**Figure 2:**
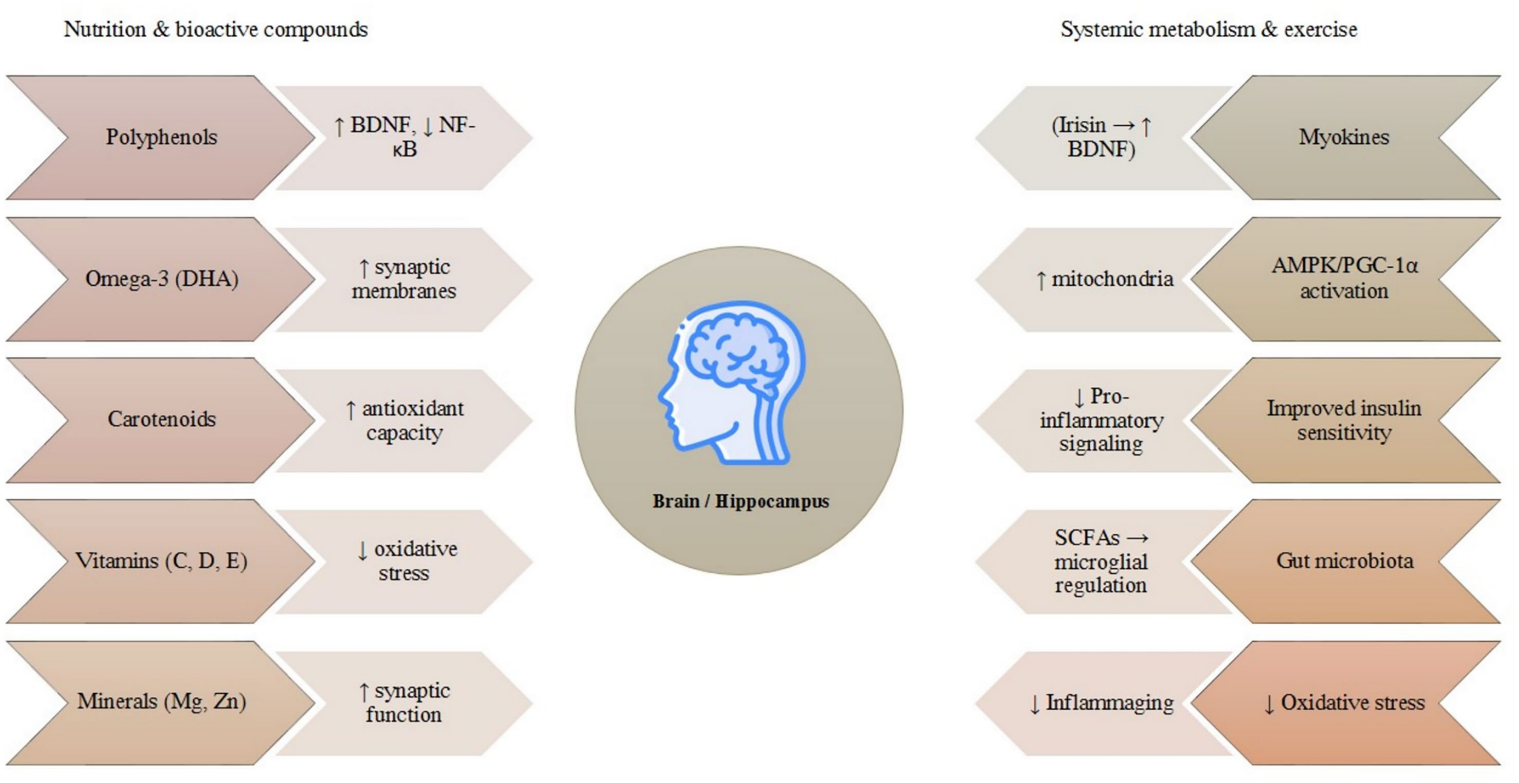
Core cellular and molecular mechanisms driving brain aging. A simplified overview of the major interlinked processes that underlie cognitive aging. Mitochondrial dysfunction increases oxidative stress, which promotes chronic neuroinflammation, leading to synaptic impairment and ultimately cognitive decline. These mechanisms reinforce one another, forming a self-perpetuating cycle that contributes to age-related vulnerability of the brain.

Aging is characterized by gradual declines in cognitive abilities, heightened neuroinflammatory responses, metabolic irregularities, and compromised hippocampal neurogenesis, all of which collectively contribute to the deterioration of memory and an increased susceptibility to *NDs* (Figure 2). Although lifestyle modifications, particularly those pertaining to nutritional adjustments and physical activity, have independently exhibited neuroprotective and metabolic advantages, recent findings underscore that their integrative or synergistic effects may exert significantly greater impacts on cerebral health. Specific classes of dietary bioactive compounds, particularly polyphenols, flavonoids, and long-chain omega-3 fatty acids, demonstrate antioxidant, anti-inflammatory, and neurotrophic effects relevant to brain health. Grasping this synergistic relationship is imperative, as it can inform the formulation of comprehensive preventive and therapeutic approaches aimed at safeguarding memory and learning amid the aging process, mitigating disease burden, and fostering healthy longevity. It should be noted that most evidence for synergistic effects currently derives from animal models, and human interventional studies remain limited in number and methodological consistency.

### 5.1 Omega-3 fatty acids

Although omega-3 fatty acids such as DHA and EPA demonstrate substantial neurobiological relevance, their bioavailability varies considerably depending on chemical form, delivery system, and dietary context (44). The absorption efficiency differs between triglyceride, phospholipid, ethyl ester, and free fatty acid formulations, with certain structures facilitating improved micelle formation and intestinal uptake. Co-ingestion with dietary fat enhances digestion and incorporation into chylomicrons, while emulsification and microencapsulation technologies have been developed to optimize bioaccessibility and stability during digestion. Recognizing these formulation-dependent differences is essential when evaluating clinical outcomes, as variability in bioavailability may partly explain inconsistent findings across nutritional intervention studies (44).

Omega-3 fatty acids, particularly DHA, are integral to neurohealth, and their insufficiency is implicated in the cognitive decline associated with advancing age. An investigation was conducted to evaluate the impact of physical exercise and two varying dosages of omega-3 supplementation (160 mg/kg and 320 mg/kg) on anxiety-like behaviors and cognitive functions in both adult and aged male rats. Seven distinct experimental cohorts were subjected to trials with or without the inclusion of exercise and omega-3 supplementation. The results indicated that the aging process adversely affects both locomotor and exploratory behaviors. In the adult cohort, the combination of low-dose omega-3 and exercise yielded a decrease in locomotor activity, whereas the administration of high-dose omega-3 alongside exercise resulted in diminished anxiety levels and enhanced memory performance. These divergent findings underscore the necessity for additional research in this domain (45) (Table 1).

**Table 1:** Synergistic effects of bioactive compounds and exercise in aging.

Evidence level and translational relevance	Kind of nutrition intervention	Duration	Model	Main effects	Mechanism of action	Ref
Preclinical mechanistic evidence	Low-dose Omega-3 (160 mg/kg) + Exercise	7 weeks	*In vivo* – Adult and Aged Male Wistar Rats	↓ Locomotor activity (adults) → Anxiety-like behavior → Recognition memory	- Limited DHA incorporation - Weak modulation of neurotransmission - Insufficient anti-inflammatory or neuroplastic response	(45)
Preclinical mechanistic evidence	High-dose Omega-3 (320 mg/kg) + Exercise	7 weeks	*In vivo* – Adult and Aged Male Wistar Rats	↓ Anxiety-like behavior ↑ Recognition memory → Locomotor activity	- Increased neuronal DHA levels - Reduced neuroinflammation - Enhanced synaptic plasticity - Synergy with exercise-induced neurotrophic factors (e.g., BDNF ↑)	(45)
Preclinical mechanistic evidence	Magnesium supplementation + Treadmill exercise	Not specified	*In vivo* – Male Albino Rats (aged)	↑ Memory performance (Y-maze and novel object recognition) ↑ Hippocampal synaptophysin (SYP) and PCNA ↓ GFAP ↓ Caspase-3 ↓ Plasma CRP ↓ Brain malondialdehyde ↑ Brain catalase	- Upregulation of hippocampal synaptic proteins (SYP) and neurogenesis marker (PCNA) - Downregulation of astrocyte activation (GFAP) and apoptosis (caspase-3) - Antioxidant effects (↑ catalase, ↓ MDA) - Anti-inflammatory effects (↓ CRP) - Synergistic effect of Mg2 + with exercise enhances neuroplasticity and cognitive function	(56)
Preclinical mechanistic evidence	Diphenyl diselenide (1 ppm) + Swimming Exercise (20 min/day, 1% body weight)	4 weeks	*In vivo* – Aged Male Wistar Rats (27 months)	↑ Hippocampal neuroprotective proteins ↓ Apoptosis-related proteins ↓ Neuroinflammation	- Selenium compound provides antioxidant and neuroprotective effects - Exercise enhances hippocampal neuroplasticity - Synergistic effect reduces apoptosis and inflammation - Combined intervention promotes hippocampal neuroprotection and cognitive support	(53)
Preclinical mechanistic evidence	Green tea supplementation + Treadmill exercise	3 months	*In vivo* – Male Wistar Rats (9 months)	↑ Antioxidant defenses ↓ Reactive oxygen species (ROS) ↑ Memory (exercise effect only) → Additional effect of green tea on memory	- Exercise reverses age-related memory impairments via neuroplasticity and cognitive stimulation - Green tea provides antioxidant effects (↓ ROS, ↑ antioxidant enzymes) - No synergistic effect of green tea with exercise on memory observed	(54)
Human interventional evidence	Diphenyl diselenide (1 ppm) + Swimming exercise (20 min/day, 3% body weight)	4 weeks	*In vivo* – Old Male Wistar Rats (24 months)	↑ Short-term memory ↑ Long-term memory ↑ Spatial learning	- Selenium compound enhances antioxidant and neuroprotective effects - Exercise stimulates hippocampal neuroplasticity - Increased hippocampal phosphorylated CREB levels - Modulation of cAMP signaling facilitates memory formation - Akt pathway unaffected	(57)
Human interventional evidence	Whey Protein (2 × 25 g/day) + Resistance Exercise (2×/week)	12 weeks	*In vivo* – Older men (67 ± 4 years)	→ Executive function → Global cognitive function → Other cognitive domains	- Protein supplementation provides amino acids for muscle and brain function - Exercise reduces systemic inflammation (TNF-*α* ↓, IL-6 ↓) - No synergistic enhancement of cognition observed - Muscle strength and physical function may support baseline cognitive performance, but changes did not translate to cognition	(58)
Human interventional evidence	Calanus Oil (n-3 PUFA) + Exercise Program	16 weeks	*In vivo* – Older women (65–80 years)	↑ Short-term memory (exercise effect) → BDNF → Added effect of n-3 PUFA	- Exercise improves cardiovascular fitness (VO2peak ↑) and cognitive performance - n-3 PUFA supplementation did not provide additional cognitive benefit - Physical activity alone supports neuroplasticity and memory function	(46)
Human interventional evidence	LCPUFA supplementation (DHA 300 mg/day + EPA 100 mg/day + ARA 120 mg/day) + Exercise (150 min/week, resistance + aerobic)	24 weeks	*In vivo* – Elderly Japanese	→ Global cognitive function ↑ Working memory (subgroup with low SMI) ↑ Selective attention (subgroup with low SMI)	- LCPUFA may enhance neuronal membrane fluidity and neurotransmission - Exercise improves cardiovascular fitness and neuroplasticity - Synergistic effects more evident in individuals with low skeletal muscle mass - Potential improvement in executive functions via combined metabolic and synaptic effects	(47)
Human interventional evidence	Multi-ingredient supplement (1,500 mg n-3 PUFA, 30 g whey protein, 2.5 g creatine, 500 IU vitamin D, 400 mg calcium) + Resistance Exercise Training + HIIT	20 weeks (Phase 1: 8 weeks supplement only; Phase 2: 12 weeks supplement + exercise)	*In vivo* – Healthy older men (73 ± 6 years)	↑ MOCA scores ↑ Word recall (RAVLT) ↑ Reaction time (executive function task) ↑ n-3 index ↓ ARA: EPA ratio	- Multi-ingredient supplement provides combined neuroprotective and metabolic support - Exercise (RET + HIIT) enhances cardiovascular fitness and neuroplasticity - Improved n-3 PUFA bioavailability supports neuronal membrane function and cognitive performance - Synergistic effect of supplementation and exercise on composite cognitive scores	(48)
Human interventional evidence	Omega-3 supplementation (DHA 800 mg/day + EPA up to 225 mg/day) + Multidomain lifestyle intervention (exercise + nutritional and cognitive counselling)	3 years	*In vivo* – Older adults (≥70 years) with subjective memory complaints	↑ Moderate-to-vigorous PA (short-term, 6 months) ↑ Leisure-time PA (short-term and maintained partially at 2–3 years) → Non-leisure PA → Light PA	- Exercise and lifestyle counseling improve motivation and adherence to physical activity - Cognitive training may enhance planning and engagement in PA - Omega-3 supplementation alone had no effect on PA - Combination sustains short-term gains in moderate-to-vigorous and leisure-time PA	(49)
Human interventional evidence	Protein supplementation (2 × 15 g/day) + Resistance-type exercise (2×/week)	24 weeks	*In vivo* – Frail and pre-frail elderly	↑ Information processing speed ↑ Attention and working memory → Episodic memory → Executive function	- Protein provides amino acids supporting neuronal function and synaptic plasticity - Exercise enhances neuroplasticity, cerebral blood flow, and cognitive stimulation - Synergistic effect particularly on information processing speed	(55)

A study assessed the efficacy of exercise, both in isolation and in conjunction with omega-3 polyunsaturated fatty acid (n-3 PUFA) supplementation, on enhancing short-term episodic memory and BDNF levels among older female subjects. Fifty-five women, aged between 65 and 80 years, participated in a 16-week regimen that incorporated circuit training and Nordic walking, during which they received either Calanus oil or a placebo. Notably, memory performance exhibited a significant enhancement post-exercise intervention across both cohorts, with no discernible advantage attributed to n-3 PUFA supplementation. The levels of BDNF exhibited no significant variation, and the observed improvements in memory were positively correlated with VO₂peak exclusively within the placebo cohort. Collectively, the results underscore the significance of physical activity in alleviating age-related cognitive decline, independent of omega-3 supplementation (46).

A randomized, single-blind, placebo-controlled investigation assessed the efficacy of integrating physical exercise with long-chain polyunsaturated fatty acids (LCPUFA) in enhancing cognitive function among people without dementia. Participants engaged in 150 min of aerobic and resistance training each week over a 24-week period while receiving either LCPUFA supplementation or a placebo; a separate non-exercise placebo cohort was utilized as a control group. Among the 76 participants who were analyzed, no statistically significant cognitive disparities were observed between the exercise and control cohorts overall. Nevertheless, subgroup analyses revealed that individuals exhibiting a low skeletal muscle mass index demonstrated enhancements in selective attention and working memory when exercise was concomitantly administered with LCPUFA. These findings suggest potential advantages of this combined therapeutic approach for older adults experiencing sarcopenia (47). Another research sought to assess the impact of multi-component nutritional supplementation, both in conjunction with and independent of exercise, on cognitive performance among older, sedentary males. A total of forty-nine subjects ingested either a nutrient-dense supplement or a maltodextrin placebo over a duration of 20 weeks, subsequently engaging in 12 weeks of resistance training and HIIT while continuing their supplementation regimen. No enhancements in cognitive function were detected during the period of supplementation alone; however, the implementation of exercise training led to significant improvements in MOCA scores, word recall, and reaction times. While intergroup differences did not reach statistical significance, it is noteworthy that only the supplementation cohort exhibited significant intragroup enhancements in composite cognitive measures. Furthermore, supplementation was associated with an increase in the n-3 index and a decrease in the ARA: EPA ratio, both of which were correlated with cognitive advancements, indicating that the combination of exercise and supplementation may confer benefits to cerebral function (48).

The Multidomain Alzheimer Preventive Trial investigated the impact of a three-year multidomain lifestyle intervention, omega-3 fatty acid supplementation, or their synergistic combination on physical activity (PA) in elderly individuals presenting with subjective memory concerns. A total of 1,680 participants aged 70 years or older were randomly assigned to one of four groups: omega-3 supplementation alone, multidomain lifestyle intervention, a combination of omega-3 and multidomain intervention, or a placebo/usual care group. Physical activity was evaluated through self-reported questionnaires that quantified moderate-to-vigorous, leisure-time, non-leisure-time, and light physical activities. The multidomain interventions resulted in a notable increase in moderate-to-vigorous and leisure-time physical activity at the six-month mark; however, these levels eventually reverted toward baseline by the conclusion of the study. Omega-3 supplementation alone exhibited no significant effects. The multidomain interventions contributed to the mitigation of long-term declines in leisure-time physical activity, underscoring the importance of integrated cognitive, nutritional, and exercise programs for the elderly population (49).

### 5.2 Polyphenols

Despite their promising neuroprotective and anti-inflammatory properties, the translational applicability of polyphenols is frequently constrained by limited oral bioavailability (50, 51). Many dietary polyphenols exhibit poor aqueous solubility, instability during gastrointestinal digestion, and extensive phase II metabolism, resulting in low systemic concentrations of parent compounds following ingestion. Additionally, interactions with food matrices and interindividual variability in gut microbiota composition significantly influence absorption and metabolic conversion into bioactive metabolites. Recent research highlights those technological approaches such as nanoencapsulation, protein conjugation, emulsification, and incorporation into optimized food matrices can enhance stability and intestinal uptake. Co-ingestion with lipids or structured delivery systems has also been shown to improve bioaccessibility and pharmacokinetic profiles (50). These findings underscore the importance of considering delivery strategies and metabolic transformations when translating polyphenol research into clinical or dietary interventions (51, 52).

The aim of a study was to ascertain whether the integration of physical exercise with a diet supplemented by diphenyl diselenide enhances neuroprotection within the hippocampus of aged rats. Male Wistar rats, aged 27 months, engaged in a regimen of daily swimming activities over a period of 4 weeks while being administered a diet containing 1 ppm of diphenyl diselenide. Analysis of the hippocampal tissue indicated that this combined intervention resulted in an elevation of neuroprotective protein levels while concurrently diminishing indicators of apoptosis and neuroinflammation. These findings demonstrated that the synergistic interplay between exercise and diphenyl diselenide supplementation may alleviate age-associated hippocampal dysfunction, thereby providing valuable insights into potential methodologies for the preservation of memory and the enhancement of cerebral health throughout the aging process (53).

A study delved into the influences of physical activity and the infusion of green tea on the biochemical and behavioral transformations associated with aging in male Wistar rats. Male rats, aged nine months, were categorized into distinct groups: a control, exercise, green tea, or a fusion of exercise and green tea, spanning a duration of three months, alongside a youthful control group for comparative analysis. Behavioral assessments revealed that engaging in physical activity countered age-related memory deficits, while green tea on its own bolstered antioxidant defenses and diminished reactive oxygen species, yet did not revive memory function. The combination of green tea and exercise failed to yield any extra advantages compared to exercise alone. These revelations underscore the cognitive enhancements brought about by physical activity in the aging process and the antioxidant prowess of green tea (54).

A secondary analysis explored the impact of a 24-week regimen of resistance-based exercises, with or without protein enrichment, on the cognitive abilities of frail and pre-frail senior citizens. Participants engaged in two exercise sessions each week or remained inactive, paired with either a protein boost (2 × 15 g/day) or a placebo. Cognitive evaluations measured various aspects including episodic memory, attention, working memory, processing speed, and executive functioning. Data revealed that the combination of exercise and protein supplementation accelerated information processing speed, whereas exercising independently bolstered attention and working memory. No noteworthy effects were detected in other cognitive areas, highlighting the specific benefits tied to these targeted interventions (55).

### 5.3 Minerals

Another work determined the efficacy of magnesium (Mg^2+^) supplementation in conjunction with treadmill exercise in mitigating age-associated memory impairments in rat models. A total of fifty male rats, categorized as young and aged, were systematically allocated into five distinct experimental groups: young, aged sedentary, aged subjected to exercise, aged receiving Mg^2+^ supplementation, and aged undergoing both interventions. The aged sedentary cohort exhibited significant memory deficits, elevated levels of C-reactive protein and malondialdehyde, diminished catalase activity, alongside notable hippocampal degeneration accompanied by altered expression profiles of synaptophysin (SYP), proliferating cell nuclear antigen (PCNA), glial fibrillary acidic protein (GFAP), and caspase-3. Both Mg^2+^ supplementation and exercise independently contributed to enhancements in memory performance and hippocampal biomarker profiles; however, the synergistic implementation of both treatments yielded the most pronounced improvements. These results suggest that the combination of Mg^2+^ and exercise acts synergistically to augment memory function through mechanisms that are anti-inflammatory, antioxidant, and neuroprotective in nature (56).

The purpose of another work was to elucidate whether the integration of a diet supplemented with diphenyl diselenide [(PhSe)₂] and swimming exercise could potentially augment memory capabilities in aged Wistar rats. Male rats aged 24 months were administered standard chow with or without 1 ppm (PhSe)₂ for a duration of four weeks, during which they engaged in daily swimming training. Cognitive performance was evaluated through the implementation of object recognition and object location assessments. The results indicated that the combined intervention significantly is associated with both short- and long-term memory, in addition to spatial learning capabilities. Mechanistically, the observed benefits were correlated with an increase in phosphorylated CREB levels within the hippocampus and alterations in cAMP concentrations, whereas Akt levels exhibited no significant changes. These findings suggest that (PhSe)₂ supplementation operates synergistically with exercise to promote hippocampal-dependent memory functions in aged rats (57).

### 5.4 Whey protein supplementation

Moreover, it is likely that resistance exercise (RE) and whey protein supplementation (PRO) may enhance cognitive performance in elderly males. In a study, 36 subjects were allocated to either the RE group or a control group with no exercise, as well as to the PRO group or a placebo supplementation group. Following a 12-week intervention, PRO was found to significantly enhance executive function and indicated a tendency toward improved global cognitive performance, while RE, whether administered alone or in conjunction with PRO, did not yield any cognitive enhancements. Nonetheless, RE was effective in diminishing inflammatory markers (TNF-*α* and IL-6); however, these alterations did not exhibit a correlation with cognitive outcomes. At the initial assessment, strength and physical function demonstrated a positive relationship with cognitive performance. In summary, PRO contributed to cognitive enhancement, while no synergistic effects were observed (58).

Evidence derived from a multitude of preclinical and clinical investigations suggests that physical exercise, whether undertaken independently or in conjunction with bioactive compounds such as omega-3 fatty acids, whey protein, magnesium, diphenyl diselenide, green tea, and comprehensive nutritional supplements, has the potential to alleviate these age-associated deficits. Mechanistically, these interventions are known to enhance synaptic plasticity and neurogenesis within the hippocampus through the upregulation of specific proteins including SYP, PCNA, and phosphorylated CREB, while concurrently diminishing apoptosis and neuroinflammation (by downregulating GFAP and caspase-3), fortifying antioxidant defenses, and modulating signaling pathways such as cAMP/CREB. Furthermore, nutritional supplementation serves to optimize the bioavailability of essential molecules like n-3 polyunsaturated fatty acids, thereby supporting cognitive functions including executive function, memory consolidation, and processing speed. Collectively, these findings imply that the synergistic integration of physical exercise and strategically selected nutrients significantly enhances cognitive performance and hippocampal plasticity in the aging population, thereby providing a mechanistic foundation for multi-domain interventions aimed at preventing age-related cognitive decline and mitigating neurodegenerative risk. It should be noted, however, that much of the evidence supporting synergistic effects in aging derives from preclinical models. Human trials examining combined exercise and bioactive compound interventions are comparatively limited in scale, often short in duration, and heterogeneous in design. Consequently, while mechanistic convergence is biologically plausible, the long-term clinical significance of these combined strategies in aging populations remains to be established through adequately powered longitudinal studies.

## 6 Synergistic effects of bioactive compounds and exercise in neurodegenerative diseases

Although the mechanistic rationale for synergy is compelling, direct clinical evidence demonstrating disease-modifying effects of combined interventions in neurodegenerative disorders remains preliminary.

### 6.1 AD

*NDs*, such as AD and PD, are delineated by a progressive decline in cognitive functions and motor abilities, alongside synaptic dysfunction, oxidative stress, and neuroinflammatory responses. The existing pharmacological interventions provide merely symptomatic alleviation, underscoring the critical necessity for preventive and adjunctive strategies. Recent investigations indicate that bioactive compounds possess the capacity to modulate molecular pathways pertinent to neuroprotection, neurogenesis, and synaptic plasticity. Furthermore, physical exercise similarly promotes the levels of neurotrophic factors, enhances mitochondrial functionality, and augments brain plasticity. The exploration of the synergistic effects of bioactive compounds in conjunction with exercise may uncover more efficacious, non-pharmacological strategies aimed at decelerating or averting neurodegeneration, enhancing cognitive and motor capabilities, and ultimately improving the quality of life for individuals afflicted by neurodegenerative conditions. The aim of study was to examine the implications of naringin administration and physical exercise on cognitive deficits and the hydrogen sulfide (H₂S) signaling pathway in rats subjected to Amyloid *β* (Aβ) injections. The rats were categorized into four distinct experimental groups: control, exercise, naringin, and a combination of naringin and exercise. The duration of the intervention spanned four weeks, encompassing treadmill exercise and the oral administration of naringin at a dosage of 80 mg/kg when applicable. The assessment of spatial learning and memory was conducted utilizing the Morris Water Maze, followed by an analysis of hippocampal biomarkers, which included S-adenosylmethionine (SAM), cystathionine-*β*-synthase (CBS), H₂S levels, and neuronal apoptosis. The results indicated that both naringin and exercise led to significant enhancements in learning and memory capabilities when compared to the control group. Furthermore, the synergistic application of naringin and exercise substantially elevated the levels of hippocampal SAM, CBS, and H₂S, implying a potential augmentation of neuroprotective effects. These findings shown that the combined effects of exercise and naringin may collaboratively enhance cognitive performance and mitigate neuronal degeneration through H₂S-mediated pathways (59) (Table 2).

**Table 2:** Synergistic effects of bioactive compounds and exercise in neurodegenerative diseases.

Evidence level and translational relevance	Kind of nutrition intervention	Duration	Model	Main effects	Mechanism of action	Ref
Preclinical mechanistic evidence	Naringin (80 mg/kg/day) + Treadmill exercise	4 weeks	*In vivo* – A*β*-injected Alzheimer’s disease model rats	↑ Spatial learning ↑ Memory performance ↑ SAM, CBS, and H2S levels ↓ Neuronal death	- Naringin acts as a neuroprotective flavonoid - Exercise enhances hippocampal neuroplasticity and cognitive function - Combined intervention increases H2S signaling pathway - Reduces neuronal death and improves synaptic function	(59)
Preclinical mechanistic evidence	Vitamin E (30 mg/kg) + HIIT (90–95% max running speed)	Not specified (acute intervention in AD rats)	*In vivo* – Male AD model rats (TMT-induced)	↑ Memory performance, ↑ SOD, ↑ PI3K, NRF2, Cat ↑ Capillary density, ↑ % healthy hippocampal cells ↑ miR-132, ↓ Amyloid beta ↓ NF-κB, ↓ MDA ↓ miR-125b	- HIIT and vitamin E synergistically activate PI3K/NRF2 antioxidant pathway - Reduce oxidative stress, inflammation, and AD markers - Enhance angiogenesis and hippocampal cell survival - Modulate miRNA expression (↓ miR-125b, ↑ miR-132) to support neuroprotection and cognitive improvement	(60)
Preclinical disease-model evidence	Postbiotic (tyndallized *Bifidobacterium longum* + *Lactobacillus acidophilus*) + High-intensity exercise	20 weeks	*In vivo* – Male APP/PS1TG transgenic mice (Alzheimer’s model)	↑ Memory performance ↑ Mitochondrial LONP1 activity, ↓ APP expression ↓ Aβ-40 plaques, ↓ NF-κB expression (exercise-specific)	- Exercise modulates NF-κB signaling and immune response - Postbiotics disaggregate amyloid-β plaques via Zn^2+^/Cu^2+^ chelation - Postbiotics decrease APP gene/protein expression - Postbiotics enhance mitochondrial protein quality control (LONP1 activity) - Synergistic intervention targets both hippocampal and extra-hippocampal regions	(61)
Preclinical mechanistic evidence	CoQ10 (50 mg/kg/day) + HIIT (4 min at 85–90% VO2max / 3 min at 50–60% VO2max)	8 weeks	*In vivo* – Male Wistar Rats, A*β*-induced Alzheimer’s model	↑ Spatial learning and memory (MWM) ↑ Recognition memory (NORT), ↑ Total thiol groups ↑ Catalase and glutathione peroxidase activities ↓ Malondialdehyde (MDA) ↓ Neuronal loss in hippocampus	- CoQ10 provides antioxidant protection, reducing oxidative stress - HIIT enhances hippocampal neuroplasticity and cognition - Combined intervention improves hippocampal oxidative status and prevents neuronal loss - Synergistic effect on cognitive functions in AD model	(62)
Preclinical neuroprotective evidence	Clove oil (0.1 mg/kg/day) + Swimming exercise (30 min/day)	3 weeks	*In vivo* – Rat model of Alzheimer’s disease (Aβ1-42-induced)	↑ Memory performance ↑ α7nAChR mRNA and protein ↓ NLRP1 mRNA and protein ↓ Dark cells	- Clove oil provides neuroprotective effects and anti-inflammatory action - Swimming exercise enhances hippocampal neuroplasticity - Combined intervention restores cholinergic function and reduces inflammasome activation - Reduces neuronal damage (dark cells) and improves cognitive function	(63)
Preclinical mechanistic evidence	L-carnosine (100 mg/kg/day) + Swimming exercise	5 weeks	*In vivo* – Rat model of Alzheimer’s disease (ICV-STZ-induced)	↑ Hippocampal FNDC5/irisin expression ↑ BDNF, ↑ Insulin signalling proteins, ↓ Soluble β-amyloid peptide ↓ Phosphorylated tau ↑ Cognitive function ↓ Hippocampal damage	- Exercise and L-carnosine enhance hippocampal FNDC5/irisin, promoting neuroprotection - Upregulation of BDNF and insulin signalling pathways - Reduction of AD pathology (β-amyloid and tau) - Both interventions ameliorate hippocampal neuronal damage and improve cognition	(64)
Preclinical evidence for mitochondrial and anti-apoptotic effects	Clove oil (0.1 mg/kg/day) + Swimming exercise (30 min/day)	3 weeks	*In vivo* – Rat model of Alzheimer’s disease (Aβ1-42-induced)	↑ Spatial memory ↓ Apoptosis ↑ PRDX6 and GCN5L1 mRNA and protein ↑ Mitochondrial homeostasis	- Clove oil provides antioxidant and neuroprotective effects - Swimming exercise enhances hippocampal neuroplasticity - Combined intervention restores oxidative balance and mitochondrial function - Reduces neuronal apoptosis and improves cognitive function	(65)
Preclinical gut–brain interaction evidence	Probiotics (*Lactobacillus plantarum* + *Bifidobacterium bifidum*) + Moderate-Intensity Interval Training (treadmill, 5 days/week)	8 weeks	*In vivo* – Rat model of Alzheimer’s disease (Aβ1-42-induced)	↑ Short-term memory ↑ BDNF mRNA ↑ ChAT ↓ Hippocampal dark cells	- Probiotics modulate gut microbiota and reduce neuroinflammation - Exercise enhances hippocampal neuroplasticity and neurotrophic factor expression - Combined intervention protects neurons, improves cholinergic signaling, and supports cognitive function	(66)
Preclinical mechanistic evidence	Probiotics (*Bifidobacterium bifidum* + *Lactobacillus plantarum*) + Treadmill Exercise	8 weeks	*In vivo* – Rat model of Alzheimer’s disease (Aβ1-42-induced)	↑ Spatial learning ↑ Acetylcholine (ACH) ↑ VEGF ↓ Hippocampal dead cells	- Probiotics modulate gut microbiota and reduce neurotoxicity - Exercise enhances hippocampal neuroplasticity - Combined intervention increases ACH signaling and VEGF expression, protecting neurons and improving cognition	(67)
Preclinical antioxidant–exercise interaction evidence	Quercetin + Treadmill Exercise	21 days Quercetin + 60 days treadmill	*In vivo* – STZ-induced Alzheimer’s disease in adult male Wistar rats	↑ Spatial memory ↓ Oxidative stress	- Quercetin acts as antioxidant, reducing ROS - Exercise enhances antioxidant defense system - Combined treatment synergistically improves hippocampal function and memory	(68)
Preclinical disease-model evidence	EGCG (50 mg/kg/day) + Voluntary Wheel-Running Exercise	4 months	*In vivo* – TgCRND8 Alzheimer’s disease mice	↑ Spatial learning ↑ Nest-building behavior ↓ Hyperactivity ↓ Soluble Aβ1-42 in cortex and hippocampus	- EGCG acts as antioxidant and reduces Aβ accumulation - Exercise improves cognitive behavior, reduces hyperactivity - Combined intervention enhances neuroprotection and behavioral outcomes	(69)
Preclinical mechanistic evidence	Naringenin (80 mg/kg/day) + Aerobic Exercise (5x/week)	4 weeks	*In vivo* – Male rats, Aβ1-42-induced AD	↑ Learning and spatial memory ↑ Hippocampal adiponectin	- Modulation of hippocampal adiponectin signaling - Synergistic effect of exercise + naringenin on memory improvement	(70)
Preclinical neuroprotective and antioxidant interaction evidence	Ecdysterone (10 mg/kg/day) + HIIT Exercise	8 weeks	*In vivo* – Male rats, Aβ-induced AD	↑ Learning and memory ↑ Exploratory behavior ↓ Anxiety-like behavior ↓ Neuronal loss	- Amelioration of hippocampal oxidative stress (↑ TAC, ↑ GPx; ↓ TOS, ↓ MDA) - Neuroprotection of hippocampal neurons - Synergistic effect of HIIT + Ecdy	(71)

A work examined the impacts of HIIT and vitamin E (VE) on the PI3K/NRF2 antioxidant signaling cascade within the hippocampal region of rats exhibiting AD, which was induced by trimethyltin (TMT). Fifty aged rats diagnosed with AD were categorized into five distinct groups: TMT control, VE solvent (sham), VE (30 mg/kg), HIIT, and HIIT+VE; additionally, ten healthy rats were utilized as a control group. The results indicated that HIIT, VE, and HIIT+VE significantly diminished levels of amyloid-beta and markers of oxidative stress (MDA), while concurrently increasing superoxide dismutase (SOD) activity and enhancing cellular health within the hippocampus. Notably, the HIIT+VE group exhibited a marked upregulation of PI3K, NRF2, and catalase expression, alongside an increase in miR-132 levels, a downregulation of miR-125b, and an augmentation of capillary density within various hippocampal regions. Enhancements in memory function were most pronounced in the HIIT and HIIT+VE cohorts. These data indicated that the combination of HIIT and VE operates synergistically to activate the PI3K/NRF2 pathway, fosters angiogenesis, mitigates the pathological features of AD, and improves neuroprotection and cognitive abilities in the hippocampus (60). Another study examined the neuroprotective influences of high-intensity training alongside postbiotic supplementation in APP/PS1 transgenic mice, which serve as a model for AD. A total of thirty-two adult mice were allocated into control, exercise, postbiotic, and combined intervention groups over a span of 20 weeks. The exercise regimen significantly diminished the expression of genes associated with AD (NF-κB), thereby modulating immune and inflammatory pathways, while the postbiotic intervention, consisting of tyndallized *Bifidobacterium longum* and *Lactobacillus acidophilus*, facilitated the disaggregation of amyloid-*β* (Aβ-40) plaques through Zn^2+^ and Cu^2+^ chelation, resulting in a reduction of APP gene and protein expression, as well as an enhancement of mitochondrial LONP1 activity, thereby improving the quality control of proteins. The synergistic application of both interventions yielded complementary advantages, wherein exercise mitigated neuroinflammation and postbiotics alleviated amyloid accumulation while bolstering mitochondrial functionality. These outcomes underscore the presence of distinct yet synergistic mechanistic pathways through which exercise and postbiotic supplementation contribute to the preservation of cognitive function and the attenuation of AD progression (61).

Another study assessed the neuroprotective effects of coenzyme Q10 (CoQ10) and HIIT, both individually and in conjunction, on cognitive deficits and hippocampal oxidative stress in Aβ-induced models of AD utilizing rat subjects. Ninety male Wistar rats were systematically allocated to diverse treatment and control groups over a duration of eight weeks. The administration of Aβ resulted in impairments in spatial learning and recognition memory, a diminution in antioxidant defenses (total thiols, catalase, glutathione peroxidase), an elevation in malondialdehyde levels, and the induction of hippocampal neuronal loss. Subsequent treatment with CoQ10, HIIT, or their synergistic application demonstrably enhanced cognitive performance as assessed by the Morris water maze and novel object recognition paradigms, reinstated oxidative homeostasis, and mitigated neuronal loss. These results imply that CoQ10 and HIIT act synergistically to confer protection against Aβ-induced cognitive deficits by augmenting hippocampal antioxidant capacity and curtailing neurodegeneration, thereby underscoring their potential as adjunctive therapeutic strategies in the context of AD (62). AD is marked by diminished expression of hippocampal α7 nicotinic acetylcholine receptors (α7nAChR) and heightened activity of the NLRP1 inflammasome, which collectively contribute to cognitive impairments and neuronal impairment. The study examined the influence of aquatic exercise and clove supplementation on these variables within an Aβ1-42-induced rat model of AD. A total of forty-eight rats were systematically allocated into six distinct groups and subjected to daily swimming sessions lasting thirty minutes and/or administered 0.1 mg/kg of clove supplementation over a duration of three weeks. Induction of AD resulted in a notable reduction in α7nAChR expression and cognitive performance, accompanied by an elevation in NLRP1 levels and an increase in dark cell counts. Both the implementation of exercise and the administration of clove supplementation alleviated these alterations, leading to enhancements in memory, an increase in α7nAChR expression, and a decrease in NLRP1 activation and neuronal injury. These outcomes exhibited that the combination of physical exercise and clove supplementation provides neuroprotective benefits in AD through the modulation of cholinergic signaling pathways and inflammasome activity (63).

Recent empirical findings indicate that irisin may operate as a neurokine, facilitating neuroprotective mechanisms. The purpose of study was to determine the impact of aquatic exercise and L-carnosine supplementation on hippocampal irisin levels and AD–like pathophysiology in streptozotocin (STZ)–induced memory-impaired rodents. The subjects received intracerebroventricular STZ injections and were subsequently allocated to either swimming, L-carnosine (100 mg/kg/day), or control groups for a duration of five weeks. Both intervention strategies reinstated hippocampal FNDC5/irisin expression, diminished soluble *β*-amyloid and phosphorylated tau concentrations, enhanced BDNF and insulin signaling proteins, and alleviated cognitive deficits. Serum and cerebrospinal fluid irisin levels remained unchanged and exhibited no correlation with hippocampal irisin. Histological assessments corroborated the reduction of hippocampal damage. The results suggested that both exercise and L-carnosine equivalently counteract cognitive deficits in AD-like scenarios, potentially through the upregulation of hippocampal FNDC5/irisin, augmentation of insulin signaling pathways, and neuroprotection against AD pathophysiology (64). AD is defined by cognitive deficits, mitochondrial dysfunction, and oxidative stress imbalance. Another research evaluated the impact of endurance training and clove oil supplementation on spatial memory, apoptosis, and mitochondrial equilibrium in a rat model of AD. Eighty-one rats were systematically allocated into nine distinct groups, encompassing various combinations of healthy, AD, exercise, and clove oil treatment conditions. The induction of AD was achieved through the intracerebral injection of Aβ1–42 into the hippocampal CA1 region. The subjects engaged in daily aquatic exercise (30 min) and/or clove oil supplementation (0.1 mg/kg) over a duration of three weeks. The onset of AD resulted in diminished expression levels of PRDX6 and GCN5L1, an elevation of apoptosis, and a decline in spatial memory performance. Both endurance training and clove oil, either in isolation or in combination, effectively reinstated the levels of PRDX6 and GCN5L1, mitigated apoptosis, and enhanced spatial memory capabilities. The data indicated that exercise and clove oil work in concert to bolster mitochondrial homeostasis, decrease apoptosis, and augment cognitive function in models of AD (65).

Dysbiosis of gut microbiota is implicated in the pathogenesis of AD through the facilitation of pro-inflammatory cytokine synthesis. The aim of work to assess the neuroprotective efficacy of moderate-intensity interval training (MIIT) in conjunction with probiotics (specifically *Lactobacillus plantarum* and *Bifidobacterium bifidum*, collectively referred to as BROB) within a rat model representative of AD. A cohort of forty male Wistar rats was administered Aβ1-42 to elicit the AD phenotype and subsequently stratified into five distinct groups: control, Aβ, Aβ + MIIT, Aβ + BROB, and Aβ + MIIT + BROB. The MIIT regimen was conducted five times per week across a duration of eight weeks, while probiotic supplementation was provided on a daily basis. The results indicated that the induction of AD led to significant hippocampal cell loss and neurodegenerative processes. The concurrent application of MIIT and probiotic treatment yielded improvements in short-term memory, enhanced expression levels of BDNF and choline acetyltransferase, and a more pronounced reduction in neuronal apoptosis compared to the effects of either intervention in isolation. The results underscored the potential synergistic neuroprotective properties of exercise and probiotics in alleviating the pathological manifestations associated with AD (66).

A study examined the impact of an eight-week regimen of treadmill exercise in conjunction with probiotics (*Bifidobacterium bifidum* and *Lactobacillus plantarum*) on cognitive capabilities and neuroprotection in a rodent model of AD. A total of twenty-five Wistar rats were allocated into five distinct groups: control, AD, AD + exercise, AD + probiotics, and AD + exercise + probiotics. The induction of AD was accomplished through the administration of Aβ1-42, and the duration of the interventions was maintained for a period of eight weeks. The assessment of spatial learning was conducted utilizing the Morris Water Maze, while measurements of hippocampal acetylcholine (ACH) and vascular endothelial growth factor (VEGF) were performed via quantitative polymerase chain reaction and immunohistochemical techniques. The results indicated that AD-afflicted rats exhibited an elevation in *β*-amyloid plaque accumulation, a decrease in ACH and VEGF levels, and a deterioration in memory function. Notably, both exercise and probiotics administered independently led to improvements in ACH levels and reductions in neuronal apoptosis, whereas their combined application yielded further enhancements in spatial learning and neuroprotective effects. These findings imply that the integration of exercise with probiotics exerts a synergistic effect that alleviates cognitive decline associated with AD (67). Another study examined the synergistic effects of quercetin, a naturally occurring polyphenolic compound, and physical exercise on cognitive deficits and oxidative stress in a rat model of AD. Fifty-six adult male Wistar rats were systematically allocated into eight distinct groups, including control, AD, quercetin-treated, exercise, and a combined quercetin plus exercise group. The induction of AD was achieved through the administration of intracerebroventricular STZ injection. Quercetin (80 mg/kg) was delivered via intraperitoneal injection over a duration of 21 days, while treadmill exercise was conducted for 1 h per day over a span of 60 days. The results indicated that STZ administration led to impairments in spatial memory and an elevation in hippocampal oxidative stress levels. Both quercetin and exercise, when applied individually, yielded improvements in memory performance and antioxidant defenses; however, their combined application demonstrated superior efficacy. These results imply that the concurrent application of quercetin and exercise preconditioning synergistically enhances antioxidant defense mechanisms and alleviates cognitive deficits associated with AD (68).

AD represents a neurodegenerative disorder that is intrinsically linked to the aging process, characterized by significant impairments in memory and cognitive faculties. Epidemiological research substantiates the notion that physical exercise and dietary antioxidants possess the capacity to diminish the risk of developing AD, with botanical flavonoids demonstrating consistently favorable protective effects. An investigation explored the synergistic effects of long-term voluntary wheel-running exercise and the oral administration of the green tea catechin (−)-epigallocatechin-3-gallate (EGCG, 50 mg/kg/day) on behavioral outcomes and Aβ pathophysiology in TgCRND8 murine models. Untreated Tg mice exhibited behaviors indicative of hyperactivity, suboptimal nest construction, and deficits in spatial learning capabilities. Both EGCG and physical exercise, whether assessed independently or in conjunction, enhanced nest building and performance in the Barnes maze, while concurrently decreasing the levels of soluble Aβ1-42 in the cortical and hippocampal regions. These outcomes shown that physical activity and dietary polyphenols work in concert to bolster cognitive functioning and alleviate AD-associated neuropathology, thereby underscoring their potential utility in the prevention of AD (69).

AD represents a predominant contributor to dementia, characterized by a scarcity of effective therapeutic interventions. Non-pharmacological strategies, including physical exercise and the consumption of flavonoids, have exhibited promising advantages. The aim of study was to determine the impact of a four-week regimen of aerobic exercise coupled with Naringenin supplementation on hippocampal adiponectin concentrations and cognitive function in Aβ1-42-induced AD rat models. A cohort of thirty-two male rats was stratified into four groups: AD, AD + exercise (ADET), AD + Naringenin (ADN), and AD + exercise + Naringenin (ADETN). Behavioral assessments indicated notable enhancements in learning and spatial memory within the ADET, ADN, and ADETN cohorts in comparison to the AD group. Furthermore, hippocampal adiponectin levels demonstrated significant augmentation, with the ADETN group exhibiting the most elevated concentrations. The findings demonstrated that both exercise and Naringenin independently promote cognitive function through the modulation of adiponectin, whereas their combined application yields a synergistic effect on memory enhancement in AD rat models (70). Hippocampal oxidative stress plays a pivotal role in the behavioral deficits associated with AD. Ecdysterone (Ecdy), a naturally occurring steroid, demonstrates notable antioxidant and neuroprotective effects, while HIIT is known to enhance cognitive function. The purpose of work to evaluate the individual and synergistic effects of HIIT and Ecdy on cognitive and behavioral impairments in rats subjected to Aβ-induced AD. Adult male rats underwent HIIT and/or received Ecdy (10 mg/kg/day) over a duration of eight weeks, commencing ten days post-Aβ administration. Behavioral assessments indicated that Aβ exposure resulted in deficits in learning, memory, exploration, and an elevation in anxiety levels, alongside hippocampal oxidative stress and neuronal degeneration. Both HIIT and Ecdy were effective in restoring oxidative homeostasis, ameliorating anxiety-like behaviors, and averting neuronal damage. Notably, the combination treatment yielded the most pronounced enhancements, implying that HIIT and Ecdy collaboratively alleviate AD-related cognitive and histological impairments through antioxidative and neuroprotective pathways (71). Overall, recent preclinical investigations elucidate that physical exercise, whether performed independently or in conjunction with natural compounds and probiotics, manifests neuroprotective properties in models of AD through the modulation of oxidative stress, mitochondrial functionality, neuroinflammation, and critical molecular pathways. Aerobic exercise and flavonoids such as naringin, naringenin, quercetin, EGCG, and coenzyme Q10 have been demonstrated to enhance spatial learning, memory, and hippocampal synaptic functionality, frequently mediated by antioxidant signaling pathways (H2S, PI3K/NRF2), elevated levels of BDNF, and diminished concentrations of Aβ and phosphorylated tau. HIIT along with vitamin E or ecdysterone has further facilitated cognitive recuperation by stimulating angiogenesis, promoting the expression of antioxidant enzymes, and enhancing neuronal viability. The modulation of gut microbiota through the application of probiotics (*Lactobacillus plantarum* and *Bifidobacterium bifidum*) has been shown to synergistically interact with exercise to elevate ACH levels, optimize mitochondrial function, and facilitate amyloid clearance. Interventions involving swimming or treadmill exercise, coupled with L-carnosine, clove oil, or postbiotics, have normalized hippocampal FNDC5/irisin and α7nAChR expression, as well as mitochondrial homeostasis, consequently leading to a reduction in apoptosis, dark cell formation, and oxidative damage. Across models, combined treatments consistently produced stronger improvements than monotherapies, suggesting synergistic mechanisms that involve modulation of insulin signaling, oxidative balance, mitochondrial quality control, neurotrophic support, and anti-inflammatory pathways. These findings collectively support the potential of integrated non-pharmacological strategies like exercise plus natural compounds or probiotics, to ameliorate AD-related cognitive deficits and neuropathological changes, providing a basis for translational studies targeting preventive and adjunctive therapies in human AD.

### 6.2 PD

The integration of physical activity and bioactive dietary constituents is increasingly recognized as a promising approach for modifying disease progression and modulating symptoms in PD, with preclinical investigations and a growing body of clinical research indicating complementary and potentially synergistic mechanisms. Physical exercise alone demonstrates neuroprotective properties in PD models and individuals diagnosed with PD by enhancing neurotrophic support, optimizing mitochondrial function, facilitating autophagy, decreasing neuroinflammation, and ameliorating both motor and non-motor functions; a substantial portion of these effects seems to be partially mediated by muscle-derived factors (exerkines) such as irisin and by the enhancement of systemic metabolic health (40, 72). In a separate context, bioactive compounds including omega-3 fatty acids, carotenoids, vitamins, and various nutraceuticals have exhibited antioxidant, anti-inflammatory, mitochondrial-stabilizing, and epigenetic effects in PD models that alleviate *α*-synuclein toxicity, safeguard dopaminergic neurons, and enhance both motor and cognitive outcomes (22, 73). When integrated, physical exercise and bioactive compounds target intersecting pathological nodes associated with PD and may yield both additive and synergistic advantages. From a mechanistic perspective, physical exercise elevates both central and peripheral BDNF levels and activates metabolic sensors that enhance mitochondrial biogenesis and cellular resilience; numerous bioactive compounds similarly engage identical or convergent signaling pathways, thereby augmenting the effects of exercise on neuronal survival and synaptic plasticity (74, 75). In preclinical investigations, the administration of dietary polyphenols or anthocyanins sourced from berries in conjunction with voluntary physical exercise results in a greater enhancement of neurogenesis markers and motor function than either intervention administered in isolation, while simultaneously diminishing microglial activation and reducing oxidative damage within the nigrostriatal pathways (76). Similarly, the combination of omega-3 fatty acid supplementation and exercise enhances mitochondrial respiratory function and attenuates inflammatory cytokine levels in preclinical models of PD, indicating complementary mechanisms that converge upon the maintenance of mitochondrial integrity and the regulation of neuroimmune responses (77). In a preclinical study using a 6-hydroxydopamine (6-OHDA) rat model of PD, the neuroprotective effects of curcumin supplementation combined with aerobic exercise were evaluated over an 8-week intervention period (78). Animals received curcumin at a dose of 100 mg/kg/day administered orally, while the exercise intervention consisted of structured treadmill running performed five days per week at moderate intensity. The study reported that 6-OHDA lesioning increased oxidative stress markers and *α*-synuclein expression while reducing antioxidant enzyme activity and dopaminergic neuron survival in the substantia nigra. Both curcumin supplementation and aerobic exercise independently improved antioxidant capacity and behavioral outcomes (78); however, the combined intervention produced greater reductions in oxidative damage and improved neuroprotective markers compared with single treatments. These findings suggest that combined nutritional and exercise strategies may exert additive or complementary neuroprotective effects through modulation of oxidative stress pathways, although translation to clinical populations requires further investigation (78).

In a preclinical study investigating combined lifestyle interventions in a PD model, Castro et al. evaluated whether blueberry juice (BBJ), a polyphenol-rich intervention, could enhance exercise-induced neuroprotection in rats exposed to intrastriatal 6-hydroxydopamine (6-OHDA) (76). Animals underwent voluntary wheel running for a total of four weeks before and after neurotoxin administration, while BBJ was provided as a dietary intervention through continuous consumption in the drinking solution. Exercise alone significantly reduced amphetamine-induced rotational behavior and attenuated dopaminergic neuron loss in the substantia nigra, whereas BBJ supplementation alone did not produce significant neuroprotective effects. However, the combined intervention resulted in greater improvements in behavioral outcomes and nigrostriatal dopamine neuron preservation compared with exercise alone. Mechanistically, exercise increased striatal glial cell line–derived neurotrophic factor (GDNF) levels, while the combination of exercise and BBJ was associated with enhanced nigral GDNF expression, suggesting that polyphenol intake may potentiate exercise-induced neuroprotective pathways through neurotrophic factor modulation (76).

Clinical evidence aligns with the plausibility of underlying mechanisms. Randomized controlled trials and studies have indicated that omega-3 supplementation may enhance depressive symptoms, inflammatory markers, and certain motor outcomes in PD, while observational and interventional exercise studies demonstrate improvements in motor scores, gait, balance, and various non-motor domains; nevertheless, the number of trials specifically evaluating combined exercise and nutraceutical interventions in PD is limited and exhibits considerable heterogeneity in design (79–81). Several pilot studies and lifestyle medicine programs imply that multicomponent approaches may decelerate patient-reported disease progression and enhance quality of life; however, larger, prolonged, and biomarker-driven randomized controlled trials are required to establish genuine disease-modifying effects and to elucidate optimal dosing, timing, and target populations (82). Several mechanistic and practical considerations dictate the extent to which synergy may be actualized in clinical contexts. Initially, the aspects of bioavailability and pharmacokinetics are of paramount importance: numerous polyphenolic compounds exhibit restricted cerebral penetration unless administered through optimized formulations or conveyed via whole-food matrices that influence the gut microbiome; physical exercise itself modifies gut transit times and microbiome composition, which may subsequently alter metabolite production and bioactive conversion interactions at the gut–brain interface (22, 40). Furthermore, the significance of timing and hormesis cannot be overstated: exercise incurs temporary oxidative and metabolic stress that catalyzes adaptive responses; excessive antioxidant supplementation may attenuate these adaptive signals, potentially diminishing the advantageous neuroplastic effects of exercise unless administered in a carefully calibrated manner concerning dosage and timing (74). Lastly, the heterogeneity of patient populations will influence their responsiveness and necessitates the implementation of stratified or personalized intervention frameworks.

From a translational lens, the most pragmatic near-term strategy involves the implementation of sustained, individualized exercise regimens for individuals diagnosed with PD, concurrently promoting dietary practices abundant in whole-food sources of neuroprotective bioactive compounds or meticulously chosen supplements with established safety profiles as adjunctive measures. Such integrative strategies present minimal risk, are consistent with overarching cardiometabolic health recommendations, and may offer symptomatic relief and potentially decelerate disease progression while more rigorous trials are undertaken (80, 81). High-priority research initiatives should encompass factorial randomized controlled trials (exercise × specific bioactive), extended follow-up periods to evaluate disease progression, and comprehensive biomarker panels aimed at elucidating the effects of peripheral interventions on central pathophysiology. In conclusion, convergent mechanistic evidence derived from preclinical studies, along with an increasing body of clinical data, substantiates the hypothesis that bioactive compounds and physical exercise may exhibit synergistic effects in the context of PD by collaboratively enhancing mitochondrial function, mitigating neuroinflammation, augmenting neurotrophic signaling, and modulating the gut–brain axis. This notion is biologically plausible and holds considerable promise; however, conclusive evidence demonstrating clinically significant modification of the disease through combined interventions remains contingent upon the execution of larger, rigorously controlled, and biomarker-enriched trials that meticulously address variables such as dosing, timing, bioavailability, and patient stratification (72, 80).

Despite encouraging preclinical findings, clinical investigations directly evaluating combined exercise and bioactive compound interventions in AD remain limited. Most available data are derived from animal models, and existing human studies are typically underpowered and short-term. Therefore, the proposed disease-modifying implications should be considered hypothesis-generating rather than clinically established.

## 7 Impact on memory and learning

Hippocampal neurogenesis and synaptic plasticity constitute essential mechanisms that underpin the processes of memory consolidation, learning, and cognitive adaptability. The aging process and *NDs*, particularly AD and PD, are correlated with diminished neurogenesis, disrupted synaptic restructuring, and impairments in LTP, culminating in a gradual decline in cognitive functions (83, 84). Preclinical investigations reveal that bioactive substances, such as polyphenols, flavonoids, omega-3 fatty acids, and carotenoids, are associated with markers consistent with hippocampal neurogenesis and synaptic plasticity via the modulation of critical molecular signaling pathways, including BDNF/TrkB, PI3K/Akt/mTOR, AMPK/PGC-1α, and CREB (see Section 4) (85, 86). Such interventions are effective in mitigating oxidative stress and neuroinflammation, thereby providing neuroprotection and facilitating the processes of synaptic remodeling (22). In a similar vein, engagement in physical exercise, most notably aerobic and resistance training, facilitates the process of neurogenesis within the hippocampus, enhances the density of dendritic spines, and augments LTP through the upregulation of neurotrophic factor expression and the optimization of mitochondrial function (87, 88). Integrative approaches that combine physical exercise with bioactive compounds have demonstrated synergistic effects in animal models, yielding more significant enhancements in spatial memory and learning capabilities than either intervention executed in isolation (68, 89). Empirical research involving older adults and individuals experiencing mild cognitive impairment indicates that comprehensive exercise and nutritional strategies can lead to improvements in cognitive performance, attention, and memory, albeit the evaluation of mechanistic biomarkers such as hippocampal volume or BDNF levels is infrequently conducted (90, 91). These observations lend credence to the notion that an approach targeting the neuro-nutritional-metabolic axis represents a promising avenue for enhancing hippocampal functionality and alleviating cognitive decline associated with aging and pathological conditions.

However, many human studies evaluating combined interventions include small sample sizes and limited follow-up durations, and mechanistic biomarkers such as hippocampal imaging or neurotrophic signaling indices are infrequently incorporated. These limitations restrict causal inference regarding sustained structural brain changes.

## 8 Limitations and future perspectives

Despite the existence of encouraging preclinical findings, empirical investigations that directly assess the synergistic impacts of bioactive compounds and physical exercise on hippocampal neurogenesis and cognitive functionality within aging or neurodegenerative cohorts are notably scarce. The majority of human trials predominantly concentrate on either nutritional interventions or exercise in isolation, frequently employing generalized cognitive evaluations in lieu of mechanistic biomarkers such as neurotrophic factors, synaptic proteins, or neuroimaging modalities that examine hippocampal architecture and functionality. Furthermore, the typical sample sizes tend to be relatively small, the durations of interventions are often truncated, and the variability in participant characteristics, stages of disease, and protocols for intervention further complicates the interpretation of the data. These constraints highlight the urgent necessity for more extensive, longitudinal, and methodologically rigorous clinical trials that assess both cognitive outcomes and the underlying molecular mechanisms. Individual variations in responses to dietary and exercise interventions can be attributed to a multitude of genetic, epigenetic, metabolic, and lifestyle factors.

A major limitation across the reviewed literature is the inconsistent assessment of mechanistic biomarkers directly reflecting hippocampal plasticity. Structural and molecular indices such as hippocampal volume, circulating or central BDNF levels, and related neurotrophic or metabolic markers are infrequently measured, particularly in human interventional studies. This gap constrains mechanistic interpretation and limits the ability to establish translational continuity between preclinical findings and clinical outcomes.

Moreover, many human trials are limited by small sample sizes, short intervention durations, and insufficient longitudinal follow-up, thereby constraining statistical power and limiting conclusions regarding sustained cognitive or disease-modifying effects. An additional translational challenge concerns the bioavailability of several bioactive compounds discussed herein, particularly polyphenols such as resveratrol and curcumin, which exhibit limited oral absorption, rapid phase II metabolism, and low systemic bioavailability. These pharmacokinetic constraints may substantially attenuate central nervous system exposure and thereby limit clinical efficacy despite promising mechanistic effects observed *in vitro* and in animal models. Emerging strategies to enhance bioavailability include nano-encapsulation, liposomal or phytosomal formulations, co-administration with lipid-rich food matrices, and modulation of gut microbiota–mediated biotransformation. Future trials should incorporate pharmacokinetic profiling and optimized delivery systems to ensure adequate target engagement in human populations.

Importantly, preventive and therapeutic applications of combined exercise and bioactive compound strategies should be conceptually distinguished. In cognitively healthy or at-risk aging populations, the primary objective is preventive: preserving hippocampal plasticity, maintaining metabolic flexibility, attenuating inflammaging, and reducing long-term risk factors associated with neurodegenerative disease. In this context, sustained moderate-to-vigorous physical activity combined with diets naturally rich in polyphenols, omega-3 fatty acids, and anti-inflammatory nutrients may represent a pragmatic and low-risk long-term strategy. In contrast, in individuals with established neurodegenerative disorders such as AD or PD, interventions function as therapeutic or adjunctive approaches, where the goals shift toward symptom mitigation, stabilization of functional decline, and enhancement of quality of life. In these populations, exercise prescriptions may require supervision and disease-stage adjustment, and bioactive supplementation may need careful consideration of dosage, bioavailability, and potential interactions with pharmacological treatments. Recognizing these distinct objectives improves translational clarity and supports the development of stage-specific, multimodal intervention frameworks rather than a uniform synergistic model.

Future investigations ought to incorporate personalized methodologies that customize the supplementation of bioactive compounds and exercise routines according to individual metabolic characteristics, nutritional patterns, and cognitive functioning. Sophisticated instruments such as nutrigenomics, metabolomics, and wearable activity trackers can enhance the effectiveness, adherence, and safety of interventions, ultimately improving neurocognitive outcomes in the contexts of aging and pathological conditions. Focusing on the neuro-nutritional-metabolic interface represents a promising direction for precision interventions. Translational research that connects preclinical insights with clinical implementation is essential for discerning effective combinations of bioactive substances and exercise strategies. By harnessing mechanistic knowledge derived from studies on molecular signaling, neurogenesis, and neural plasticity, forthcoming strategies could yield customized, multimodal interventions that mitigate cognitive decline, bolster memory and learning capacities, and augment overall cerebral resilience in the context of aging and *NDs*.

## 9 Conclusion

In this narrative review, we elucidated the synergistic interactions between bioactive compounds and physical exercise on hippocampal neurogenesis, synaptic plasticity, and cognitive functioning in the context of aging and neurodegenerative pathologies. Interpretation of the findings should consider the limited number of human trials directly testing combined interventions and the variability in mechanistic biomarker assessment, as discussed in Section 8.

Across the studies summarized in Tables 1, 2, the temporal coordination between bioactive compound supplementation and exercise interventions was rarely designed as an independent experimental variable. In most preclinical models, supplementation was administered chronically (daily oral gavage, dietary enrichment, or intraperitoneal injection) throughout the intervention period, while exercise protocols were conducted concurrently over several weeks. Similarly, human trials typically implemented long-term supplementation alongside structured training programs without systematically aligning supplement intake with specific exercise sessions or acute post-exercise windows. Consequently, although several investigations reported additive or synergistic outcomes on cognitive performance, neurotrophic signaling, oxidative balance, or mitochondrial function, these effects cannot be attributed to optimized timing strategies. Importantly, none of the reviewed studies directly compared pre-exercise versus post-exercise supplementation, nor did they evaluate whether temporal alignment with exercise-induced metabolic or redox signaling influenced adaptive neuroplastic responses. Given the hormetic nature of exercise-induced oxidative and inflammatory signaling, the timing of antioxidant or metabolic supplementation may critically determine whether interactions are synergistic, neutral, or potentially attenuating. Therefore, while concurrent administration appears biologically plausible and often beneficial, the concept of “temporal synergy” remains insufficiently investigated and represents a significant methodological gap requiring rigorously controlled, time-stratified intervention designs in future research.

Across the studies, exercise interventions varied in modality and intensity, yet several consistent patterns emerge. In preclinical models of aging and neurodegeneration, structured aerobic paradigms, most commonly treadmill running or swimming performed 5 days per week for 3–8 weeks, were the predominant approach and were frequently associated with improvements in spatial memory, antioxidant capacity, neurotrophic signaling, and reductions in apoptosis. HIIT protocols, although less frequently used, appeared to induce robust effects on oxidative balance, angiogenesis, and molecular signaling pathways in AD models. Resistance-type paradigms were less represented in animal studies but showed cognitive benefits in human aging cohorts, especially when performed 2–3 times per week over 12–24 weeks. Importantly, most synergistic effects with bioactive compounds were observed under conditions of regular, repeated training rather than acute or short-duration exposure, suggesting that cumulative metabolic and neurotrophic adaptations are critical for interaction effects. Overall, the evidence supports sustained, moderate-to-vigorous aerobic exercise as the foundational modality for synergistic strategies, while higher-intensity or resistance-based programs may provide additional domain-specific benefits depending on age, disease status, and functional capacity. However, direct comparative trials examining dose–response relationships remain limited, underscoring the need for standardized exercise prescriptions in future multimodal intervention studies.

Regarding bioactive compound dosing, investigations demonstrate substantial heterogeneity in administered amounts, with most preclinical investigations employing doses that fall within experimentally established safety ranges but are often higher (on a body-weight basis) than typical human dietary exposure. For example, omega-3 fatty acids were administered in animal models at approximately 160–320 mg/kg, whereas human trials generally used nutritional-range supplementation consistent with commonly accepted safe intakes (e.g., ≤3 g/day combined EPA/DHA). Polyphenols such as naringin, naringenin, quercetin, EGCG, and diphenyl diselenide were typically delivered in the range of 50–100 mg/kg in rodents, doses that are considered non-toxic in experimental settings but exceed habitual dietary intake and may not directly translate to humans without formulation adjustments. Vitamin E (e.g., 30 mg/kg in animal models) and coenzyme Q10 were administered within ranges below known toxicity thresholds, though high-dose antioxidant supplementation in humans must be carefully monitored due to potential pro-oxidant or hormetic interference effects. Compounds such as L-carnosine (100 mg/kg), ecdysterone (10 mg/kg), magnesium supplementation, probiotics, clove oil (0.1 mg/kg), whey protein (e.g., 15–30 g/day in human studies), and postbiotics were likewise used within reported safety margins in both animal and clinical contexts. Importantly, most human interventions utilized doses consistent with established tolerable upper intake levels or clinically accepted supplemental ranges, suggesting general safety under controlled conditions. Nevertheless, direct comparisons across studies are complicated by differences in bioavailability, formulation, route of administration, and species-specific metabolism. Moreover, few investigations systematically evaluated dose–response relationships or optimized dosing in relation to exercise intensity and timing. Therefore, while the reviewed evidence suggests that synergistic benefits occur within commonly accepted safety ranges, further rigorously designed, dose-ranging clinical trials are necessary to determine the precise effective and optimal dosing strategies for each bioactive compound when combined with structured exercise interventions.

Preclinical and nascent clinical evidence suggests that integrated interventions modulate critical molecular pathways, thereby facilitating neuronal survival, synaptic reorganization, and enhancements in memory and learning capabilities. Although solitary interventions involving diet or exercise yield significant benefits, their synergistic combination appears to confer additive effects, thereby optimizing hippocampal functionality and alleviating cognitive deterioration. These observations accentuate the promise of combined lifestyle-oriented strategies as a non-pharmacological methodology for the preservation of cognitive health and the attenuation of neurodegenerative disease progression. Nonetheless, the current corpus of clinical evidence is still constrained, particularly in relation to individualized regimens, optimal dosing parameters, and long-term ramifications. Importantly, while preclinical studies and early clinical findings suggest potential synergistic benefits, no definitive evidence currently demonstrates disease-modifying effects of combined exercise and bioactive compound interventions in humans with AD, PD, or other neurodegenerative conditions. Therefore, the translational implications discussed herein should be interpreted as biologically plausible and hypothesis-generating rather than clinically established. Moreover, the majority of human studies to date are small-scale and relatively short in duration, and thus do not yet provide robust longitudinal evidence for sustained clinical benefit or disease modification.

Future investigations should prioritize meticulously designed clinical trials that encompass mechanistic biomarkers, neuroimaging techniques, and tailored intervention strategies. Furthermore, the incorporation of translational insights derived from preclinical investigations can inform the formulation of precision nutrition and exercise regimens that specifically address the neuro-nutritional-metabolic axis. These methodologies exhibit potential for the postponement or mitigation of cognitive deterioration, the enhancement of memory and learning capabilities, and the augmentation of overall cerebral resilience among aging individuals and those afflicted with *NDs*.
